# Meningitis caused by *Pasteurella multocida* in a dog owner without a dog bite: clonal lineage identification by MALDI-TOF mass spectrometry

**DOI:** 10.1186/s13104-015-1615-9

**Published:** 2015-10-31

**Authors:** Matthieu Bardou, Estelle Honnorat, Gregory Dubourg, Carine Couderc, Pierre Edouard Fournier, Piseth Seng, Andreas Stein

**Affiliations:** Service de maladies infectieuses, Hôpital de la conception, Assistance publique-hôpitaux de marseille, 147, Boulevard baille, Marseille, France; Aix-Marseille Université, URMITE, UM 63, CNRS 7278-IRD 198, INSERM 1095, Faculté de Médecine, 27 Bd Jean Moulin, 13385 Marseille, France

**Keywords:** The matrix-assisted laser desorption/ionization-time of flight mass spectrometry, MALDI-TOF, Clonal lineage, Typing, *Pasteurella multocida*, Meningitis, Infection, Bacteria, Human

## Abstract

**Background:**

*Pasteurella multocida* meningitis in an immunocompetent patient is rare and commonly occurs after animal bite. To our knowledge, only 48 cases have been reported in the literature since 1989. *P. multocida* meningitis is commonly linked to animal contagion. Here we report on a new case of *P. multocida* meningitis in an immunocompetent patient who is a dog owner without a dog bite. We used the matrix-assisted laser desorption/ionization-time of flight (MALDI-TOF) mass spectrometry to investigate the clonal lineage between animal and human isolates.

**Case presentation:**

In our case, a 25-year-old immunocompetent French Caucasian woman with nothing notable in her medical history was admitted for meningitis caused by *P. multocida*. Clonal lineage of *P. multocida* strains from cerebrospinal fluid and blood culture and her dog’s oral cavity has been recognized by MALDI-TOF mass spectrometry dendrograms and clustering of the 21 *P. multocida* isolates in our centres. She was treated by a combination of intravenous ceftriaxone (2 g/day) and oral levofloxacin (1 g/day). She was discharged on the 6th day of admission. The antimicrobial therapy was conducted for 15 days. The dog was treated by clavulanic-acid amoxicillin for 3 weeks by the veterinarian. The evolution of the patient at the 5th month after the end of the antimicrobial therapy was normal without any neurological after-effects.

**Conclusion:**

The meningitis caused by *P. multocida* could be considered a cause of human meningitis in dog lovers without an animal bite. MALDI-TOF mass spectrometry should be considered as it is an accurate tool to identify clonal lineage between animal and human isolates.

## Background

*Pasteurella multocida* are small Gram-negative coccobacilli which appeared to be a common flora in oral cavity of dogs and cats [1]. Skin and soft tissue infection is a main human pasteurelloses; and *P. multocida* meningitis in an immunocompetent patient is rare and commonly occurred after animal bite [2–5].

Species identification of *Pasteurella* in routine clinical microbiology laboratories is mainly based on convention phenotypic identification. The variation of phenotypic characteristic between isolates from different hosts may results in imprecise species identification and needs molecular identification such as 16S rRNA gene sequence analysis in these cases [6]. Matrix-assisted laser desorption/ionization-time of flight (MALDI-TOF) mass spectrometry has been recently introduced to clinical microbiology laboratories for accurate bacterial species identification [7]. Zangenah et al. have recently reported an advantage of MALDI-TOF mass spectrometry in *P. multocida* comparing to conventional microbiological methods and VITEK^®^ 2 microbial identification system (bioMérieux) [8].

Here we report on a rare case of *P. multocida* meningitis in an immunocompetent dog owner without a dog bite. We used MALDI-TOF mass spectrometry to investigate the clonal lineage between animal and human isolates.

## Case presentation

In January 2015, a 25-year-old French Caucasian woman was admitted to the infectious disease department of the university hospital in Marseille for a headache, nasal congestion and vomiting. Prior to the headache, she had been fine with nothing notable in her medical history. She lives with a dog and she had not travelled to a tropical area. We did not find any recent indication of an animal bite or a skin wound in her medical history. On admission, she had a high fever (40.3 °C), her pulse was 117 beats/min, and her blood pressure was 138/41 mmHg. The clinical examination showed a subarachnoid syndrome and frontal-occipital headache. Laboratory investigations revealed an elevation of the C-reactive protein level (275 mg/L; normal < 5 mg/L), an elevation of the plasmatic fibrinogen level (6.82 g/L; normal values = 1.8–4 g/L), an elevated leukocyte count (13,000/µL), predominantly neutrophil granulocytes, a low lymphocyte level (430 µL; normal = 1500–4000 µL) involving T cells, although B and NK cells, a normal haemoglobin concentration (14.8 g/dL), and a low platelet count (148 × 10^3^/µL; normal = 150–400 × 10^3^/µL). Cerebrospinal fluid sample (CSF) analysis revealed an elevated protein level of 2 g/dL, a low glucose level of 74 mg/dL (plasmatic glucose level = 189 mg/dL) and a WBC count of 1200 cells/mm^3^ with 90 % neutrophils. CSF cultures and blood cultures revealed positive for *P. multocida* as identified by MALDI-TOF mass spectrometry. Laboratory tests including complement and immunoglobulin analysis did not find immunodeficiency.

She was treated by an empirical intravenous antimicrobial therapy with a combination of cefotaxime (4 g/day), amoxicillin (12 g/day) and acyclovir (2 g/day), the antimicrobial therapy was modified for a combination of intravenous ceftriaxone (2 g/day) and oral levofloxacin (1 g/day). The patient improved rapidly with a disappearance of the subarachnoid syndrome at 2 days of treatment but the fever persisted. A cerebral magnetic resonance imaging (MRI) did not reveal any abnormality and she became afebrile only on the 4th day of antibiotic treatment. She was discharged on the 6th day of admission. The antimicrobial therapy was conducted for 15 days. The evolution at the 5th month after the end of the antimicrobial therapy was normal without any neurological after-effects.

**Fig. 1:**
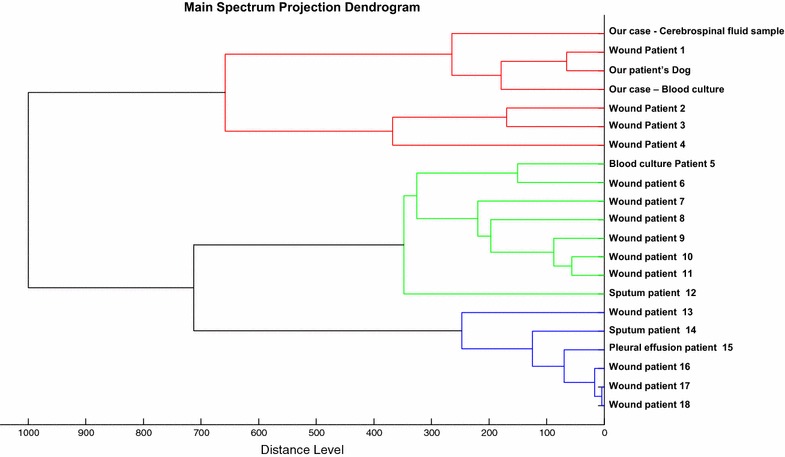
The matrix-assisted laser desorption/ionization-time of flight mass spectrometry mass spectrometry dendrograms and clustering of *P.* *multocida* isolated from CSF and blood culture of our patients and *P. multocida* isolates in our centres

**Fig. 2:**
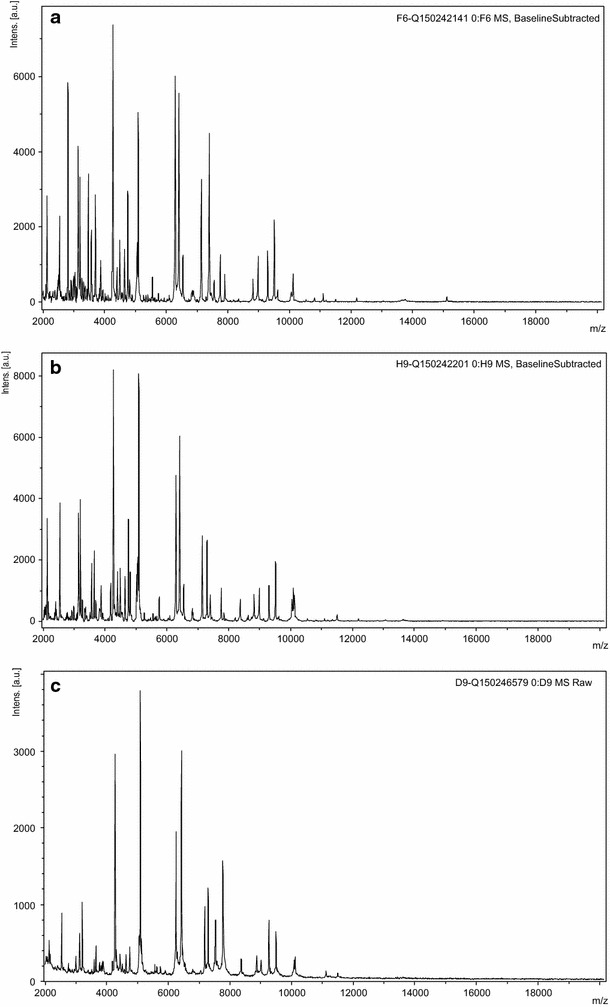
The matrix-assisted laser desorption/ionization-time of flight mass spectrometry mass spectra obtain from colonies isolated from: **a** the patient’s blood culture, **b** the patient’s cerebrospinal fluid sample (CSF), **c** the patient’s dog oral cavity

Bacterial cultures in the oral cavity of her dog tested positive for *P. multocida,* which displayed the same antibiotic susceptibility pattern than isolates from the patient’s blood and CSF. In addition, main spectrum projection (MSP) enabled to group the three strains in a same cluster as defined by an arbitrary distance level <300, indicating they were closely related (Figs. 1, 2). The dog was treated by clavulanic-acid amoxicillin for 3 weeks by the veterinarian.

## Discussion

We herein report a new case of meningitis caused by *P. multocida* in a young immunocompetent patient who was probably infected by her dog without a dog bite. In the literature, the main risk factor for *P. multocida* meningitis is an animal contact, which has been reported in 84 % of cases [2]. To our knowledge, only 48 adult cases of meningitis caused by *P. multocida* in immunocompetent and immunodeficiency patients have been reported [2–5]. Only nine cases of neurological complications have been reported including epidural empyema, cerebrals abscess, convulsion, cognitive deficit, paralysis, acute disseminated encephalomyelitis and meningoencephalitis [2–5, 9]. According to previous studies, risk factors for *P. multocida* meningitis include age >55 years old [2] and alcoholism [9].

In 26 % of cases a medical past-history of recent neurosurgical surgery was found [2]. In our case, the patient had no apparent immunodeficiency factors and no neurological surgery. She was discharged and cured after 15 days of antibiotic treatment with ceftriaxone 2 g/day and levofloxacin 1 g/day. In the literature, antibiotic treatment with intravenous betalactamin with median duration of 21 days (range: 10–27 days) has been usually reported [2]. Nevertheless, ten of the 48 reported cases of *P. multocida* meningitis died.

Human pasteurellosis is not frequent. Some cases of human pasteurellosis have occurred in animal lovers by kissing pets [10, 11] and pigs [5]. However the carriage rate of *P. multocida* in an animal’s oral cavity is high (12–75 %) [1]. Pulsed field gel electrophoresis (PFGE) by using 2-D gel electrophoresis and molecular analysis have long been considered as the gold standard for clonal analysis. These methods have been used in the studies of 117 porcine and human isolates of *P. multocida* [12], and 23 field and vaccine strains [13]. In the last decades, MALDI-TOF mass spectrometry identification become a routine method in clinical laboratories [7]. In some condition, this technique has been used to identify clonal lineage bacteria in previous studies [7, 14–16]. In our case, the *P. multocida* strains of the patient and her dog were revealed to be closely related as identified MALDI-TOF mass spectrometry.

## Conclusion

The meningitis caused by *P. multocida* could be considered a rare cause of human meningitis that can be occurred in immunocompetent patient without an animal bites or scratches. In the alternative of molecular identification, MALDI-TOF mass spectrometry is a rapid and accurate identification method of *P. multocida* isolates. The clonal lineage characterization between animal and human isolates in our study was possible using routine MALDI-TOF mass spectrometry.

## Consent

This study was approved by the institutional research ethics board (Comite de Protection des Personnes Sud Méditerranée 1), and written informed consent was obtained from the patient for publication of this case report and any accompanying images. Written informed consent was obtained from the patient to take samples from her dog.

## Abbreviations

*P. multocida*: *Pasteurella multocida*
MALDI-TOF mass spectrometry: the matrix-assisted laser desorption/ionization-time of flight mass spectrometry
CSF: cerebrospinal fluid sample
PFGE: pulsed field gel electrophoresis
MSP: main spectrum projection
NK cells: natural killer cells
